# Well-Preserved Structure of Silicified Wood: A Case Study from Qitai Silicified Forest, NW China and Its Silicification Mechanisms

**DOI:** 10.3390/plants14223468

**Published:** 2025-11-13

**Authors:** Wenqing Liu, Guanghai Shi, Xinling Li, Xiaoyun Quan, Yuetong Li, Ye Yuan

**Affiliations:** 1 State Key Laboratory of Geological Processes and Mineral Resources, China University of Geosciences, Beijing 100083, China; liuwenqingcugb@foxmail.com (W.L.); quanxiaoyun@grinm.com (X.Q.); lixiaotong327@foxmail.com (Y.L.); yuenyeah@163.com (Y.Y.); 2 Xinjiang Uygur Autonomous Region Production Quality Supervision and Inspection Institute, Urumqi 830013, China; lixinling001@foxmail.com; 3 Youke Publishing Co., Ltd., Beijing 100088, China

**Keywords:** silicified wood, wood anatomy, mineralogical characterization, conifer wood microstructure, wood preservation

## Abstract

The Qitai silicified wood from Xinjiang, NW China, provides an exceptional archive for investigating the mechanisms of wood silicification. This study applies microscopy, scanning electron microscopy (SEM), and X-ray diffraction (XRD) to characterize the microstructural and mineralogical features of these fossils. The results show that the samples are primarily composed of microcrystalline–macrocrystalline α-quartz having anhedral–euhedral shapes, with minor occurrences of moganite. A well-preserved structure exhibits distinct anatomic details of cellular networks, such as growth rings and rays. Magnified observation revealed that the microcrystalline quartz within cell walls grew outward from the innermost layer of the wall, suggesting silica infiltration from lumina to walls. The opposite growth of elongated columnar quartz within adjacent cell walls terminated at the position of the middle lamellae. Cell lumen infilling exhibits greater variability on filling degree and phase type. The permeation silicification of cell walls and the oligoblastic to polyblastic structure inside cell frameworks contribute to high fidelity preservation. This interpretation helps us understand how the wood structure was perfectly preserved during the silicification, thus emphasizing its significance for wood identification through its preserved structure.

## 1. Introduction

Fossil wood, the petrified remains of ancient trees, represents one of the most captivating archives of terrestrial paleoenvironments and paleoecological history [1,2,3,4,5,6]. Among various modes of wood petrifaction, silicification stands out for its unparalleled capacity to preserve the intricate cellular and subcellular anatomical details of the original wood with exceptional fidelity [7]. Silicified wood is a complex combination of plant tissue structure and mineral microstructure, with inorganic structures superimposed on residual organic networks [8]. The cells are interconnected, acting as a grid of organic tissue passages allowing for silica solution impregnating. The anatomy of silicified wood has been widely studied to analyze plant evolution processes [3,9,10,11].

The microstructure of wood plays a decisive role in determining the pathways and mechanisms of silicification. Silicification typically occurs when wood is buried in an environment that inhibits decay, such as anoxia levels [12,13]. In addition, silicification may occur rapidly, allowing the cell structure to be preserved before it has completely decayed or collapsed [14,15,16,17,18]. The silica represented as monomeric silicic acid (Si(OH)_4_) and its derivative forms (such as H_4_SiO_4_°, H_3_SiO_4_^−^, and their oligomers and polymers), initially forms hydrogen bonds with functional groups in the cell wall and precipitates lining the cell wall which acts as a template (a process known as permineralization) [19,20]. Through the formation of siloxane bonds and loss of water, silica monomers polymerize and gradually precipitate in the form of porous silica gel through heterogeneous nucleation [19,21]. Subsequently, the infill of cell lumina and the gradual replacement of the space of cellulose and lignin in cell walls via organic degradation gradually proceed [15,22,23]. Various forms of silica, such as opal-A, opal-CT, chalcedony and crystalline α-quartz, commonly coexist inside silicified wood [22,24,25,26,27]. According to previous microstructure studies, samples from different localities have varying distributions of silica phases in their cell structures, and the silicification patterns of cell walls and cavities may be completely different. Layered preservation of cell walls and variations in silica distribution within cellular structures have been revealed on the Chemnitz petrified wood from Germany by Dietrich et al. (2013) [21]. Despite a general understanding of the silicification process, the mechanism by which wood anatomical structures are preserved with high fidelity remains an intriguing subject. Silicified wood from various localities requires further investigation to discover some characteristics or deepen the understanding of commonalities on the preservation mode of wood silicification.

The Qitai Silicified Forest (QSF) is a prominent fossil site in Xinjiang, NW China, and contains more than 1000 silicified trees, mostly in situ stumps [28]. The fossil forest is considered to have been preserved in the Late Jurassic [29]. Previous studies have identified the wood form-genera being *Protopiceoxylon*, *Xenoxylon*, *Araucarioxylon* and *Cupressinoxylon* [29,30] and used tree-ring analyses to infer paleoclimatic conditions such as drought and temperature variations [31]. Although the gemological characteristics and microstructure of one sample was initially documented by Li et al. (2016) [32], a thorough structural analysis of these specimens is still indispensable for elucidating the cellular structure preservation through silicification.

Here, we select well-preserved samples and present a detailed analysis of the anatomical features and the forms, structure, and distribution of silica in silicified wood from the QSF, providing new insights into the wood structural preservation through silicification and its fundamental significance for ancient wood identification.

## 2. Geological Setting and Sample Description

**Figure 1:**
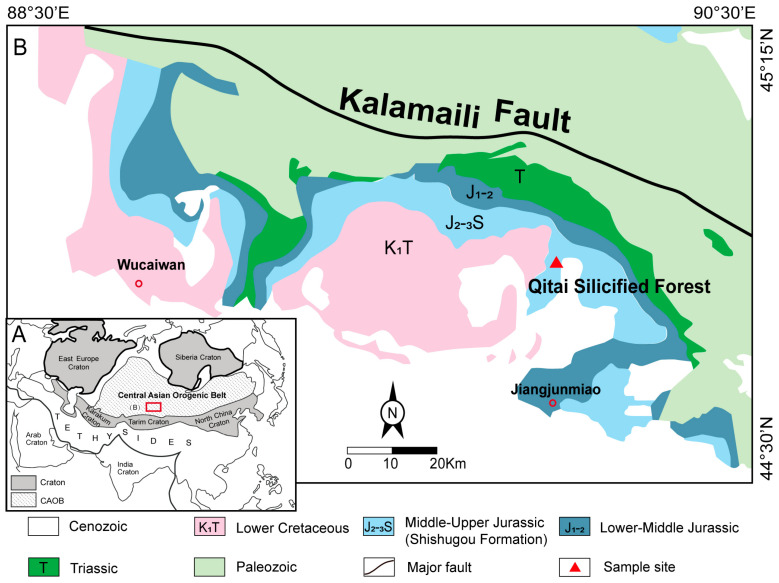
(**A**) Tectonic framework of the Altaids and adjacent regions (modified after Xiao et al. (2009) [36]). (**B**) Geological map of the South Kalamaili Range in eastern Junggar Basin, showing the sample site and stratigraphic relations. Adapted with permission from Yang et al. (2014) [37], 2025, John Wiley and Sons.

The QSF is located in Xinjiang, China, approximately 100 km northeast of Qitai County. It occurs within the eastern Shishugou Formation outcrops, exposed along the south Kalamaili Range in the eastern Junggar Basin of the southwestern Altaids (Figure 1) [33,34]. The formation strata dip between 0–10 degrees in a generally westward direction, resulting from the influence that the bordering Kalamaili Range exerted on the adjacent Jurassic strata [34]. The formation consists primarily of a basal conglomerate, interbedded mudstone and sandstone sequences, red beds, and multiple tuff layers, collectively indicative of deposition in an alluvial–fluvial floodplain environment. It was chronologically attributed to the Middle–Late Jurassic [29,33,34,35]. The silicified forest horizon lies in the sandstone-mudstone interval of the upper part of the Shishugou Formation, where ancient trees grew on the weathered crust and were covered by a layer of gray-white tuff [18].

**Figure 2:**
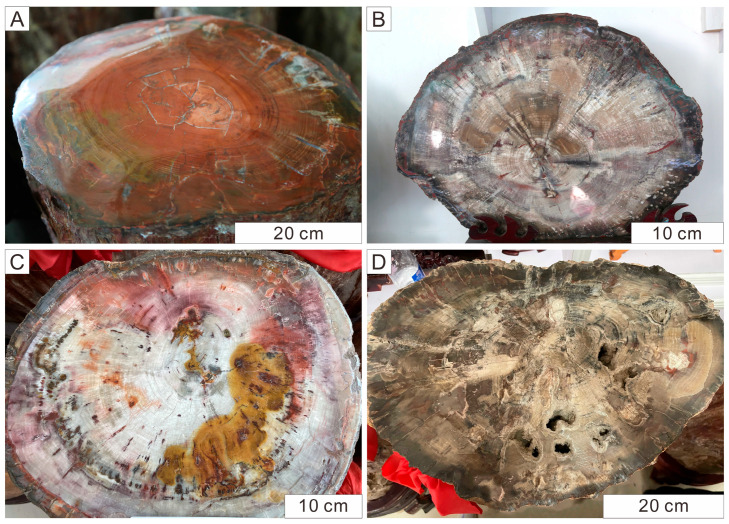
Appearance characteristics of well-preserved Qitai silicified wood. Fossil wood is mainly brownish red and brownish gray in color (**A**,**B**,**D**), with distinct color blocks showing in some stumps (**C**). Clear anatomical structures such as growth rings can be seen. Visible quartz crystal clusters grew from the inner walls of the voids (**D**). All photographs by the authors.

Silicified wood from the QSF typically exhibits the appearance and texture characteristic of coniferous wood. Clearly discriminable growth rings are displayed in the well-preserved specimens. The color is primarily grayish-brown, although more vivid specimens exhibit hues ranging from brownish-yellow to reddish-brown. Certain specimens display slight desiccation cracks likely induced by desiccation or degradation of organic matter (Figure 2) [38]. All of our samples were collected from the stumps or logs in the field of QSF. From a total collection of over 30 samples, five were selected for detailed X-ray diffraction (XRD) and scanning electron microscopy (SEM) analysis based on the clear cellular structures and the well-defined crystalline texture of the quartz infilling observed under microscope. All selected specimens we used in this study exhibit a characteristic brown color and visible wood textures.

## 3. Materials and Methods

Mineral composition of the silicified wood samples was determined using a SmartLab X-ray powder diffractometer (XRD, BRUKER AXS LIMITED, Marxzell, Germany) at the X-ray powder diffraction laboratory, China University of Geosciences (Beijing). Powdered samples (<200 mesh, ~0.5 g each) were analyzed under Cu-Kα radiation (45 kV, 200 mA) with graphite monochromation. Scans ran continuously from 3° to 70° 2θ at 8°/min under controlled conditions (25 °C, 56% RH). The obtained XRD patterns were analyzed using the reference data from the International Centre for Diffraction Data (ICDD) database. Quartz crystallinity was assessed using the five-finger diffraction peak complex (67–69° 2θ). The crystallinity index (CI) was calculated using CI = 10 × F × a/b, where a and b represent the peak height of the 67.8° 2θ (1.38Å d-spacing) diffraction peak above background level [39]. A synthetic quartz standard (CI = 10) was used for calibration, yielding F = 1.182 at a scan rate of 0.25°/min [32].

Laser micro-Raman spectroscopic analyses were performed with an HR-Evolution Micro Raman spectrometer (HORIBA JOBIN YVON, Villeneuve d’Ascq, France) at the Gemmology Experimental Teaching Centre, CUGB. A frequency-doubled Nd:YAG laser (λ = 532 nm) and a grating of 600 gr/mm was used for analysis of the silica phases of a silicified wood section. Each spot was analyzed for 15 s (five acquisitions, 3 s each) in the range of 100–900 cm^−1^. Background noises were corrected by subtracting a linear baseline separately.

The microstructural characteristics of the silicified wood samples, including the size, morphology, and distribution of quartz particles, were examined using a SUPRATM 55 field emission scanning electron microscope (FE-SEM, ZEISS, Oberkochen, Germany) at the State Key Laboratory of Biogeology and Environmental Geology, China University of Geosciences (Beijing). Freshly fractured surfaces of the samples were coated with a thin layer of platinum prior to imaging to enhance conductivity. The observations were conducted at an accelerating voltage of 10 kV, with a resolution better than 0.8 nm.

## 4. Results

### 4.1. Anatomical Structure

**Figure 3:**
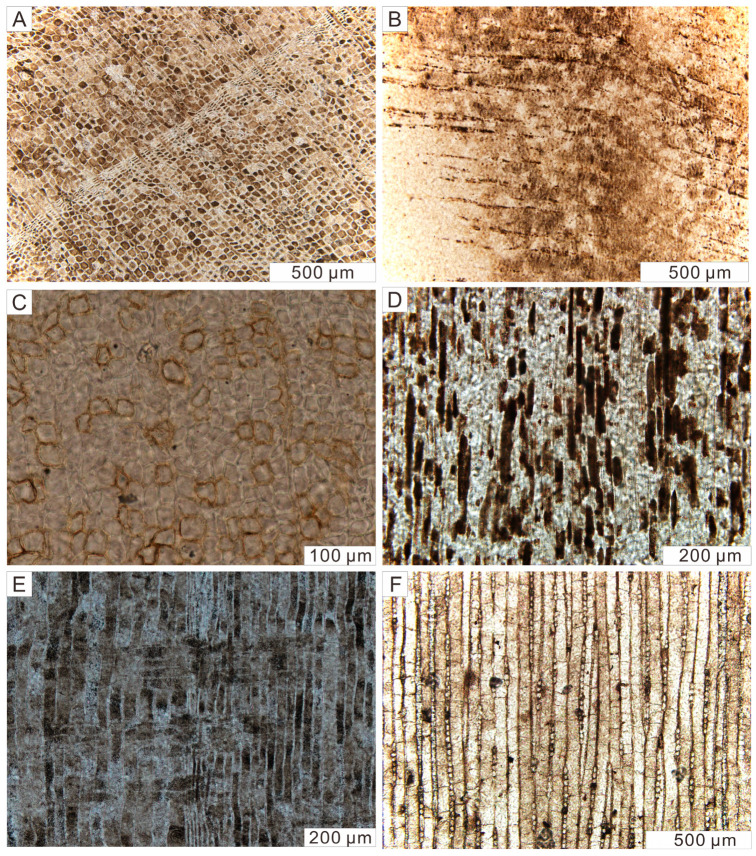
Micrographs of three-dimensional sections of well-preserved Qitai silicified wood samples. Transmitted light. (**A**) The growth ring composed of early and late wood cellular structures. Transverse section. (**B**) Wood rays outlined by dark spherical inclusions. Transverse section. (**C**) Visually discontinuous cellular structures. Transverse section. (**D**) The rectangular profile of tracheids. Radial section. (**E**) The cross-field structure formed by the intersection of tracheids and wood rays. Radial section. (**F**) Wood ray groups mixed in the middle of tracheids showing typical fusiform shapes. Tangential section.

All samples exhibit anatomical features of coniferous wood. In the transverse sections of our samples, the boundary between earlywood and latewood is distinct, showing clearly visible growth rings. Uniseriate rays are arranged perpendicular to the growth rings (Figure 3A). Occasionally, these rays contain accumulations of dark brown spherical inclusions (Figure 3B). Sometimes, brownish materials are unevenly distributed within the cell walls of densely arranged and roughly rectangular tracheids, resulting in a discontinuous appearance of the tissue (Figure 3C). In the radial section, longitudinally sectioned tracheids exhibit a roughly rectangular outline, with lengths averaging approximately 100 µm (Figure 3D). Cross fields are present, although bordered pits on the tracheid walls were not observed, possibly due to the section cutting inside the cell (Figure 3E). On the tangential section, no transverse resin canals are identified. Uniseriate rays with typically fusiform shapes are clearly visible (Figure 3F).

### 4.2. Silica Mineralogy

**Figure 4:**
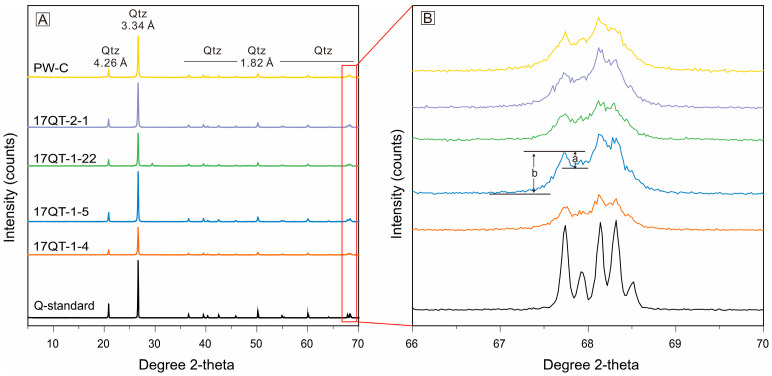
X-ray diffraction (XRD) diffraction spectra (**A**) and five finger peak spectra (**B**) of well-preserved Qitai silicified wood samples. “a” and “b” represent peak height of the 67.8° 2θ (1.38Å d-spacing) diffraction peak above background level. Abbreviation: Qtz = quartz.

All diffractograms of the Qitai silicified wood samples exhibited characteristic double-peaks of α-quartz at d _(100)_ 4.25 and d _(101)_ 3.34 (Figure 4A). The positions and intensities of the remaining diffraction peaks showed strong consistency with the data of standard α-quartz (Table 1), confirming that the dominant mineral phase is α-quartz. No additional weak reflections indicative of moganite (bimodal peaks of 4.43 Å and 3.38 Å) or opal-CT (~4.1 Å) were detected in the XRD patterns [40]. The crystallinity index (CI) of the samples, calculated using the five-finger diffraction peak method, ranged between 3.54 to 4.46 (Figure 4B, Table 1).

**Figure 5:**
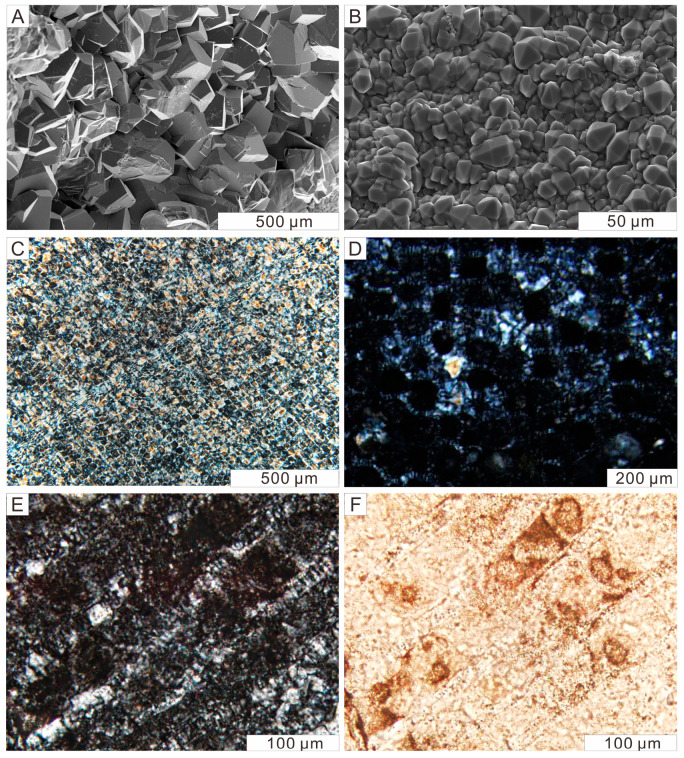
Scanning electron microscopy (SEM) and microscopic images showing morphology and types of SiO_2_ minerals within well preserved Qitai silicified wood. (**A**) Macrocrystalline α-quartz in rhombohedral crystal form (pseudocubic quartz). (**B**) Euhedral microcrystalline quartz with complete terminations showing unoriented distribution. (**C**) Randomly oriented anhedral, mosaic-like microcrystalline quartz grains filling in cellular structures. Polarized light. (**D**,**E**) Tracheid lumina filled with fine-grained, “dark” minerals. Polarized light. (**F**) Mottle areas showing superposition of opaque impurities. Transmitted light.

**Figure 6:**
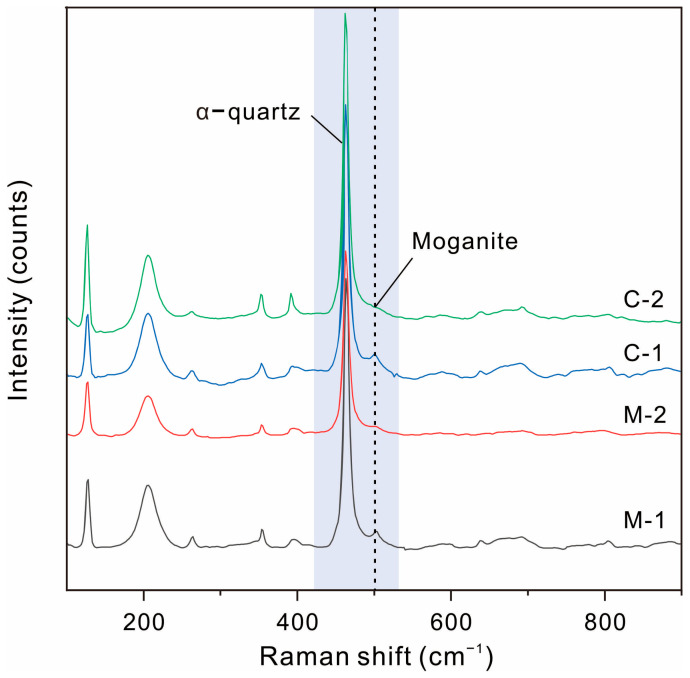
Raman spectra of fine-grained minerals in tracheid lumina of a well-preserved Qitai silicified wood sample, showing characteristic bands of α-quartz and moganite. Abbreviation: C = clean area, M = mottle area.

Macrocrystalline quartz was rarely observed in the Qitai silicified wood samples, occurring only as a late-stage crystalline phase within voids. In some cases, it grows in free space and displays a radial-oriented pattern surrounding the silicified wood matrix center, with the c-axis oriented nearly perpendicular to the center, forming external euhedral terminations [18]. Occasionally, trigonal α-quartz exhibiting a rhombohedron-dominated habit (pseudocubic quartz) is observed (Figure 5A). Microcrystalline quartz sometimes occurs as euhedral crystals with complete terminations, developing within micro-scale cellular pores. Their crystallographic orientation varies, ranging from random with no consistent alignment between adjacent crystals to perpendicular to the cell wall (Figure 5B,D,E). Commonly, it occurs as randomly oriented anhedral, mosaic-like grains, showing distinct birefringence under polarized light (Figure 5C). Typical lepispheres indicative of opal-CT were not observed by SEM. Exceptionally in a sample, the cell lumina were observed to be filled with fine-grained minerals, exhibiting overall optical darkness under cross-polarized light (Figure 5E). Areas of complete extinction, designated as “mottle areas”, were attributed to the superposition of opaque impurities by plane-polarized light confirmation (Figure 5F). In contrast, regions without such impurities were labeled “clean areas”. Raman analysis conducted on both clean and mottle areas not only revealed the predominance of α-quartz (465 cm^−1^) but also showed the weak moganite band at 501 cm^−1^ of varying intensities (Figure 6).

### 4.3. Silica Structure Inside Well-Preserved Cellular Frameworks

**Figure 7:**
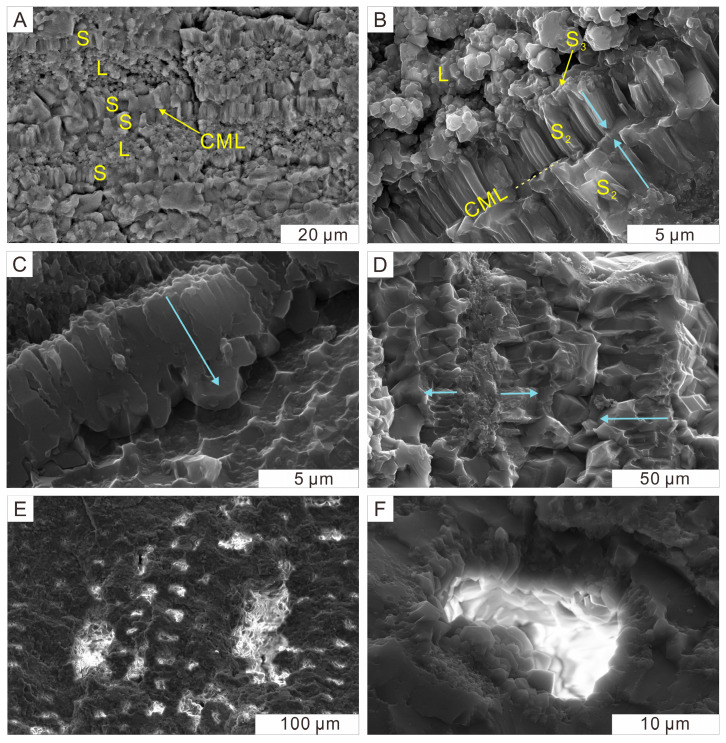
SEM images showing differential distribution of silica minerals in cellular structure of high-fidelity preservation. The blue arrow represents the growth direction of the quartz columns inside cell walls. (**A**) Elongate, columnar, subhedral microcrystalline quartz grains growing perpendicularly within the cell wall, while lumina are being filled with microcrystalline grains. Longitudinal section. (**B**) Anhedral microcrystalline quartz lining the innermost layer of cell wall, while columnar quartz is extending outward from this layer and the crystal fronts are terminating at the compound middle lamella. Longitudinal section. (**C**) The termination surface showing crystal impressions. Longitudinal section. (**D**) Divergent two to three layers of quartz crystals within cell walls. Longitudinal section. (**E**,**F**) The center of lumina being unsilicified, and the wall being lined with euhedral quartz. Transverse section. Abbreviation: S = secondary wall, L = lumen, CML = compound middle lamella, S_2_ = the middle layer of secondary wall, S_3_ = the inner layer of secondary wall.

In well-preserved samples, silica phases typically precipitate in accordance with the constraints of the precursor cellular structures (Figure 7). For instance, in a sample exhibiting well-defined growth rings and cellular structures, polarized light reveals a honeycomb-like distribution of quartz crystals mostly aligned within the cellular framework (Figure 5C). In other cases, cell walls and lumina are often accentuated by distinct silicification textures (Figure 5D and Figure 7). In the longitudinal section, microcrystalline quartz commonly occurs as elongate and columnar subhedral grains, growing radially and perpendicularly to the cell wall boundaries (Figure 7A). At higher magnification, the innermost layer of the cell wall is lined with fine anhedral quartz grains. Columnar quartz crystals extend outward from this layer, exhibiting subhedral terminations. The adjacent cell wall of another cell displays a symmetrical growth pattern, with crystal fronts terminating along the same and nearly continuous plane (Figure 7B). This contact interface may preserve crystal impressions from either side (Figure 7C). In some positions, two to three distinct layers of quartz crystals in varying lengths composing the cell wall can be observed (Figure 7D). Some tracheid lumina are densely packed with porous aggregate composed of nanoscale microcrystalline quartz and moganite particles (Figure 5D and Figure 7A,B), while some lumina remain unsilicified in the center and are lined with terminated euhedral quartz crystals projecting inward from the walls (Figure 7E,F).

## 5. Discussion

The silicified wood samples examined in this study exhibit a high degree of anatomical preservation. Tracheids display well-aligned organization and minimal evidence of deformation or shrinkage, with cell wall boundaries remaining distinct. Structures such as wood rays, cross field and legible growth rings (alternating earlywood and latewood) are also well preserved. This structural integrity suggests that silicification occurred within a relatively stable diagenetic environment, where anoxic conditions and rapid silica infiltration effectively inhibited destructive processes such as organic decay and physical compaction [12,13,14,15,16,17,18].

Since the diffraction peaks of chalcedony coincide with those of quartz, its broader reflections may be overlapped by the sharper quartz peaks, preventing definitive identification of chalcedony through XRD analyses. However, microscopic examination did not reveal any chalcedonized cell walls within silicified cellular structures, which has been reported in silicified wood from other localities [23,24]. The values of crystallinity index (3.54–4.46) fall within the typical range reported for silicified plant fossils (1.0–4.6) and are relatively high [39]. This is consistent with the crystalline characteristics observed under polarizing microscopy and SEM, which revealed predominantly euhedral to anhedral crystalline quartz. The consistent Raman signature without the opal-A broad band (400 cm^−1^) in both clean and mottle areas suggests that the optical darkness in the mottle areas arose from incorporated opaque impurities rather than opal-A [41,42]. The occurrence of moganite in silicified wood has been well-documented in specimens from various localities, including Germany, Turkey, Thailand, Myanmar, Mongolia, Oman, France and the United States [12,26,43,44]. However, as evidenced by peak assignments in previous studies [12,44], the dominant XRD diffraction peaks of quartz often overlap with those of moganite. Even with improvements in peak amplification techniques [45], XRD inherently suffers from low sensitivity to short-range order or minor phases. In contrast, Raman spectroscopy has proven to be a more reliable tool for detecting the existence and spatial distribution of the moganite component in α-quartz in silica rocks [42,45,46]. It is also crucial to note that moganite in silicified wood can form through low-temperature evaporation precipitation, hydrothermal precipitation and diagenetic transformation of silica phases [47,48,49,50,51]. Quartz has similar origins, either directly precipitating from slightly supersaturated solutions or transforming from silica polymorphs with lower order [23,24,25,44,46,49,50,52,53,54]. Considering that Qitai silicified wood remains in situ under the cover of tuff and silicified under high-temperature conditions, the silicification process may involve co- and post-volcanic hydrothermal fluids and the silica minerals initially precipitated from the fluids [18,26,43,44]. The tight combination of α-quartz and metastable silica polymorph moganite may suggest silica phase transformation in low-temperature diagenetic settings as well [23,24,25,44,46,49,50,53,54].

**Figure 8:**
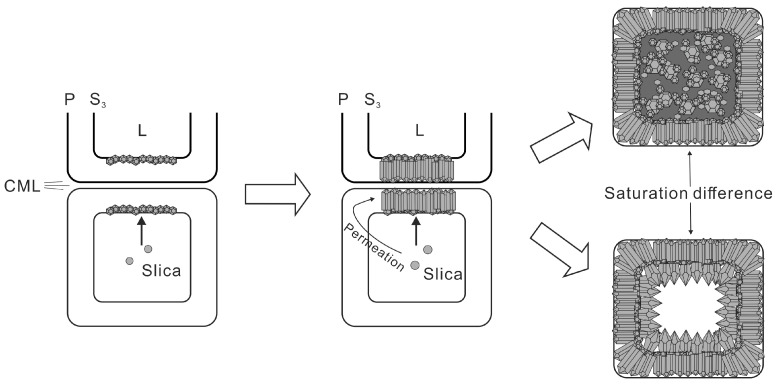
Schematic diagram of silicification mode in wood cell structure. First, the silica entering from the cell lumina preferentially precipitated on the inner wall of the cell. Second, quartz inside cell walls extended outward and terminated at the compound middle lamella. Third, the siliceous solution inside the cell lumina precipitated minerals at different supersaturations, forming different silicification patterns. Abbreviation: P = primary wall, S_3_ = the inner layer of secondary wall, L = lumen, CML = compound middle lamella.

Silicification can effectively preserve the original anatomical structure of wood, allowing for the inference of dimensional characteristics of the precursor cells from silicified specimens as well as the further clarification of wood taxonomy. Following the entry of silica into the cell lumen, precipitation preferentially occurred on the inner cell wall due to the affinity between silica and hydroxyl groups exposed by organic components [20,23,24]. As silica permeated from the lumen through the wall, quartz growth proceeded from the innermost layer of the secondary wall (S_3_) toward the outermost primary wall. The submicroscopic structure within the secondary cell wall, such as microfibrils, may affect the pathway and rate of silica infiltration, ultimately leading to the formation of crystal forms completely different from that in cell lumina. Although initial crystallization might be guided by the microfibrillar structure inside the secondary wall [21,54], crystal habit exerted a stronger influence as growth progressed. In accordance with the geometric selection law, only those crystals with the fastest-growing direction oriented perpendicular to the substrate persisted. These parallel columnar quartz crystals might have parallel overgrowth structures (Figure 8). The intact and plump cell morphology preserved by silica minerals exhibits no obvious signs of shrinkage, suggesting rapid silicification occurring potentially while the tree was still alive or shortly after its death.

Subsequent silicification of the lumen led to diverse textures due to local variations in silica saturation. In some cases, the lumen is filled with porous anhedral nanoscale particles, suggesting rapid and massive nucleation under relatively high silica saturation (Figure 5D and Figure 7A–D). Due to the possible existence of diagenetic transformation processes, the supersaturation of the solution that precipitated fine-grained silica phases might be higher than that of the current situation, resulting in the initial precipitation of lower order phases such as opal [52,53]. In other instances, the lumina remains hollow but lined with terminated euhedral quartz crystals projecting inward from the walls (Figure 7C,D). It displayed a “micro agate” structure, similar to agate geodes forming quartz clusters in the latest stage with the center possibly vacant [23,55]. Quartz precipitating slowly from weakly supersaturated solutions favored the development of highly ordered crystalline structures. It typically infilled cell lumina or voids during the later stage of a successive silicification process, reflecting reduced silica availability in the environment (Figure 7C). Additionally, the compound middle lamella (CML) between adjacent cells often remains unsilicified. After initially acting as a barrier to crystal growth from both sides, it might subsequently degrade and disappear (Figure 8).

The fidelity of preservation is strongly influenced by the structure of the silica minerals. Based on the size and abundance of SiO_2_ crystals within the wood cells, the silicification structure can be classified into oligoblastic, polyblastic, hyperblastic and idioblastic structures [56], which represent a single crystal in one cell, multiple crystals in one cell, a single crystal growing beyond the cell and euhedral crystal clusters in any part of the fossil, respectively. According to our microstructural observation, the oligoblastic and polyblastic structure (Figure 5C,D and Figure 7) typically facilitates high-fidelity preservation, whereas hyperblastic and idioblastic structures often fail to retain clear and complete anatomical details which need further elaboration thereafter.

In addition, the general absence of directional layering in the silica structures of the Qitai silicified wood as well as that in other fossil wood localities [15,23,24,25] shows analogy to the formation of agate geodes in intermediate-acidic volcanic rocks [57,58], unlike the gravity-controlled growth of layered chalcedony [59]. It suggests that silicification likely occurred under an atypical diagenetic environment, potentially independent by gravitational sedimentation. Two plausible mechanisms are proposed: (1) petrification within a high-temperature vapor stability field, where silica transport and precipitation were governed by thermal convection and vapor-phase diffusion; (2) silica fluid migration controlled primarily by capillary action. These two mechanisms may have a joint effect on multiple episodes (if they exist) of the petrification process among various localities, but not simultaneously. The first mechanism was supported by a successful wood silicification experiment under silica-rich vapor conditions [60] and the silicification temperature and pressure conditions of Qitai silicified wood inferred from inclusion analyses [18]. For the other inference, the interconnected lumina of xylem tracheids can be regarded as a network of fine capillary tubes, in which fluid adsorption and transport are governed by pore diameter, surface tension, and wettability. During early-stage wood silicification, capillary forces are considered a potential driver for the infiltration of silica sols or oligomeric silicic acid into cellular microstructures [19]. Following entry into the wood, silica-bearing fluids accumulate within these microchannels as capillary water, where surface tension enables localized fluid retention by counteracting gravitational drainage [61].

## 6. Conclusions

The Qitai silicified wood primarily consists of α-quartz with minor moganite occurrence. Initial cell-wall silicification preserving anatomical details was followed by variable lumen infilling without directional layering characteristics, reflecting non-gravitational deposition. Preservation fidelity is quite high for wood fossils with oligoblastic and polyblastic structure. This interpretation elucidates the mechanism by which the anatomical structure of wood was meticulously preserved during silicification, thereby underscoring its significance for wood identification based on well-preserved anatomical features.

## Figures and Tables

**Table 1:** Indexing results of XRD analysis and crystallinity indexes of well-preserved Qitai silicified wood samples.

Sample Parameters		Standard Quartz		17QT-1-5		17QT-1-22		17QT-2-1		17QT-1-4		PW-C	
		d/Å	I/I0	d/Å	I/I0	d/Å	I/I0	d/Å	I/I0	d/Å	I/I0	d/Å	I/I0
hkl	100	4.25	21.62	4.26	18.50	4.26	18.90	4.26	17.20	4.26	17.30	4.25	17.20
	101	3.34	100.00	3.34	100.00	3.34	100.00	3.34	100.00	3.34	100.00	3.34	100.00
	110	2.46	6.00	2.46	5.40	2.46	5.60	2.46	4.70	2.46	5.70	2.46	5.20
	102	2.28	5.90	2.28	6.10	2.28	6.60	2.28	6.10	2.28	6.50	2.28	5.90
	111	2.24	2.60	2.24	2.60	2.24	2.20	2.24	2.50	2.24	2.50	2.24	2.30
	200	2.13	4.30	2.13	3.90	2.13	4.00	2.13	3.80	2.13	3.90	2.13	4.00
	201	1.98	2.40	1.98	2.50	1.98	2.70	1.98	2.70	1.98	2.60	1.98	2.70
	112	1.82	9.60	1.82	9.20	1.82	10.10	1.82	9.60	1.82	8.80	1.82	9.20
	202	1.67	2.60	1.67	2.30	1.67	2.80	1.67	2.50	1.67	2.80	1.67	2.40
	211	1.54	5.80	1.54	5.30	1.54	5.20	1.54	5.10	1.54	5.50	1.54	5.10
	113	1.45	1.00	1.45	1.20	1.45	1.10	1.45	1.00	1.45	1.00	1.45	0.90
	212	1.38	3.20	1.38	3.50	1.38	3.30	1.38	3.40	1.38	3.50	1.38	3.90
	203	1.37	4.10	/	/	1.37	4.80	1.37	5.60	1.37	4.70	1.38	5.50
CI		10		4.453		3.706		3.544		4.456		3.936	

## Data Availability

Data are contained within the article.
